# Self perceived health and stress in the pregnancy during the COVID-19 pandemic

**DOI:** 10.3389/fpsyt.2023.1166882

**Published:** 2023-03-31

**Authors:** Cristina Liebana-Presa, María Cristina Martínez-Fernández, Rubén García-Fernández, Cristian Martín-Vázquez, Elena Fernández-Martínez, Pedro Hidalgo-Lopezosa

**Affiliations:** ^1^Department of Nursing and Physiotherapy, Universidad de León, León, Spain; ^2^SALBIS Research Group, Faculty of Health Sciences, Ponferrada, Spain; ^3^SALBIS Reseach Group, Faculty of Health Sciences, León, Spain; ^4^Department of Nursing, Pharmacology and Physiotherapy, Universidad de Córdoba, Córdoba, Spain; ^5^Maimonides Biomedical Research Institute of Cordoba (IMIBIC), Córdoba, Spain

**Keywords:** pregnancy, COVID-19, psychological distress, prenatal maternal stress, Goldberg general health questionnaire, anxiety

## Abstract

**Introduction:**

The COVID-19 pandemic has had numerous maternal and neonatal consequences, especially at the mental level. Pregnant women experience a rise in anxiety symptoms and prenatal stress.

**Aims:**

The aim was to describe self-perceived health status, general stress and prenatal stress and to analyze relations and associations with sociodemographic factors.

**Methods:**

A quantitative, descriptive and cross-sectional study was conducted using non-probabilistic circumstantial sampling. The sample was recruited during the first trimester of pregnancy during the control obstetrical visit. The Google Forms platform was used. A total of 297 women participated in the study. The Prenatal Distress Questionnaire (PDQ), the Perceived Stress Score (PSS) and the General Health Questionnaire (GHQ-28) were used.

**Results:**

Primiparas presented higher levels of worry about childbirth and the baby (10.93 ± 4.73) than multiparous women (9.88 ± 3.96). Somatic symptoms were present in 6% of the women. Anxiety-insomnia was scored positively by 18% of the women. In the Spearman correlation analysis, statistically significant values were found between almost all study variables. A positive correlation was observed between self-perceived health and prenatal and general stress levels.

**Discussion:**

During the first trimester of gestation, prenatal concerns increase when levels of anxiety, insomnia and depression also increase. There is a clear relationship between prenatal worries, anxiety, insomnia and depression with stress. Health education that focuses on mental health of pregnant women would help reduce worries during pregnancy and would improve the pregnant women perception of her health and well-being.

## Introduction

The pandemic caused by COVID-19, has impacted the health of the pregnant women (1, 2). COVID-19 pandemic was declared by the Word Health Organization (WHO) on May 11th of 2020 (3, 4), and different prenatal care restrictions emerged. Prenatal and postnatal appointments were cancelled, accompaniment throughout the entire process was restricted, the use of masks was made mandatory, and breastfeeding was even discouraged in some places (5, 6). At this time, the medium and long -term consequences of COVID-19 infection during pregnancy are still unknown (7).

The SARS-Cov-2 disease has caused an increase in anxious symptoms in the general population, especially pregnant women, due to the possible fear of becoming infected (8). In addition, the prevalence of anxiety and stress in pregnant women is higher than in the general population of women (9, 10).

Insomnia is one of the most important alterations we find in pregnant women, and its influence on mental health during pregnancy has been studied by different authors in recent years (11–13). During the COVID pandemic, an increase in the number of women with this disorder was detected (14). Research by Kendle et al. found a relationship between insomnia and the coexistence of other mental health disorders in pregnant women and a possible relationship with physical effects (13). In addition, insomnia during the perinatal stage has been shown to be closely related to the risk of depressive symptoms (11).

Women who are pregnant are perceived to be positive about the gestation process, but they also see it as a risky process, both for her and her future baby (15). Moreover, several studies have reported that high levels of maternal stress can have adverse effects on both the pregnant woman and the fetus (16, 17). It is important to investigate the possible negative effects of psychological stress during pregnancy and its relationship with the health of the pregnant woman (18).

Additionally, during the first trimester of gestation, pregnant develop specific stress associated with the prenatal stage (19). It is estimatedthat at least 25% of this population will develop this type of stress. Maternal stress during pregnancy is clearly related to some adverse outcomes in both the newborn and the pregnant woman, we can find low neurological development in newborns, low birth weight children, prematurity and more anxious pregnant women (20, 21).

On the other hand, many instruments have been used to measure this specific gestational stress, including the Prenatal Distress Questionnaire (PDQ) (22), which has been recommended for the assessment of pregnancy-related stress. It is a widely used instrument and there are versions of this instrument available in English, widely used in the United States, United Kingdom and Ireland (23), in German (24) and in the Spanish-speaking population (25), making it one of the most commonly used instruments in those population.

Different authors appear to disagree about the relationship between women’s parity and the presence of stress. Various studies have shown that having two or more previous births, is a risk factor for high levels of stress during gestation (19), while other studies have shown completely opposite results, presenting multiparity as a protective factor (26).

Women with higher general or pregnancy-specific stress scores were hypothesized to perceive poorer general health.

To know how stress influences self-perceived health during the first trimester of gestation of the COVID 19 pandemic, the objectives of this research were as follows. To describe self-perceived health status, general stress and prenatal stress and to analyze the relationships and associations with sociodemographic factors. Health promotion and prevention of maternal and neonatal consequences are essential for the well-being of a healthy and vulnerable population (pregnant women) at a time of health crisis (COVID 19 infection).

## Methods

### Study design and sample

The study was descriptive and cross-sectional. By means of a non-probabilistic circumstantial sampling, we selected pregnant women who attended the obstetrics consultation for the first time. They belonged to a Regional Management of the public health system of Castilla y León (Spain) and were in their first trimester of pregnancy. Those who had a previous diagnosis of depression, anxiety or psychiatric illness, language difficulties in the recruitment process, failure to sign the consent form or refusal to participate in the study were excluded. Medical records of the participating women were reviewed to see if they had any mental health diagnoses that would lead to exclusion from the study. During the year 2021 there were a total of 501 births in the region under study (Junta de Castilla y León, 2021), the minimum sample size required for this study was determined using a single population proportion formula with a 95% confidence level assumption, a precision (d) of 3% and an expected loss percentage of 15%, resulting in a required sample of 170 women (27).

### Procedure

Participating women were recruited during the first trimester control visit at the reference hospital. After the consultation with the obstetrician, participants were invited to fill in the questionnaire *via* the Google Forms platform by sending them an email. The completion time was 10 min and participants did not receive any incentive in return. Data were collected between September 2021 and June 2022.

### Ethical considerations

The participants gave their consent voluntarily. The protocol was reviewed and approved by both the University ethics committee and the Clinical Research Ethics Committee in accordance with the clinical research standards established by the scientific community.

### Measures

The Prenatal Distress Questionnaire (PDQ) (25) measures pregnancy-specific stress by means of 12 items. Responses are based on a 5-point Likert-type scale where 0 = not at all and 4 = very much. A maximum score of 48 can be achieved. Three factors are described “Worries about childbirth and baby,” “Worries about weight or self-image” and “Worries about emotions.” Caparros-Gonzalez et al. carried out the validation of this questionnaire for the Spanish population, obtaining a Cronbach’s alpha of 0.74 (25). Cronbach’s alpha coefficients of the subscales are 0.77, 0.86 and 0.77, respectively, (23, 28).

The Perceived Stress Scale (PSS) (29), provides information on self-perceived stress during the past month. It consists of 14 items scored on a 5-point Likert-type scale (0 = never, 1 = almost never, 2 = occasionally, 3 = often, 4 = very often), providing a maximum score of 56. The Cronbach’s alpha obtained in the validation studies was 0.81. Trujillo et al. carried out the validation of this questionnaire for the Spanish population (29).

The General Health Questionnaire (GHQ-28) (30). A 28-item scale validated by Lobo et al. (31) consisting of four subscales of seven items each measuring social dysfunction, health perception (somatic symptoms), anxiety-insomnia and major depressive symptoms during the previous 2 weeks. The traditional formula called GHQ (0,0,1,1) was used to establish the cut-off point (32). Subsequently, they were dichotomized by taking the cut-off point of 5/6 as described by Goldberg et al. in 1979 (30). Lobo et al. carried out the validation of this questionnaire for the Spanish population (31). The internal consistency measured in terms of Cronbach’s alpha in the validation study by Molina et al. showed the following values: 0.82 for somatic symptoms, 0.85 for anxiety-insomnia, 0.78 for social dysfunction and 0.88 for major depression (33).

### Data analysis

A descriptive analysis was performed using central tendency, dispersion and frequency measures. After verifying that the quantitative variables did not fit a normal distribution using the Kolmogorov–Smirnov test with the Lilliefors significance correction, nonparametric tests were performed. Therefore, the Spearman’s rho test was used to analyze the correlation coefficient to analyze the associations between quantitative variables. The relationship between quantitative and qualitative variables was determined using the Mann Whitney *U* test. To calculate the psychometric indicators of the measurement instrument used, the reliability coefficient (Cronbach’s alpha) was analyzed. Finally, simple logistic regression models were used between the dimensions of the GHQ-28 scale as dependent variables and the PDQ and PSS variables. Statistically significant results were established with a value of *p* <0.05. The SPSS v.26 statistical package was used for data analysis.

## Results

A total of 391 first trimester pregnant women agreed to participate in the study, and the response rate to the questionnaire was 75.95% (*n* = 297). The mean age was 33.61 years, with a maximum of 47 years and a minimum of 20 years, and the mean length of pregnancy at the time of assessment was 8.7 weeks (SD = 1.94). In terms of parity, 54.5% (*n* = 162) of the women were multiparous while for 45.5% (*n* = 135), it was their first pregnancy. Of the women, 92.6% were Spanish while the remaining 7.4% were foreign, 5.4% (*n* = 16) were Latin American and 2% (*n* = 6) were non-Spanish Europeans. Table 1 describes the sample according to the sociodemographic variables analyzed.

**Table 1:** Sociodemographic and obstetric characteristics of the woman in the study.

Sociodemographic variables		*n* = 297 (100%)
Parity	Primiparous	135 (45.5%)
	Mutiparous	162 (54.5%)
Marital status	Married/ cohabiting	241 (81.1%)
	Single/ widowed	56 (18.9%)
Abortions	None	209 (70.4%)
	One or more	88 (29.6%)
Cesarean	Non	255 (85.9%)
	One or more	42 (14.1%)
Childbirth	None	206 (69.4%)
	One or more	91 (30.6%)
Area of residende	Rural	88 (29.6%)
	Urban	209 (70.4%)
Tipeo f pregnancy	Spontaneus	277 (93.3%)
	Assisted reproduction	20 (6.7%)
Nationality	Spanish	275 (92.6%)
	Inmigrant	22 (7.4%)

Table 2 shows the descriptive statistics by measures of central tendency of stress (PSS), prenatal concerns (PDQ) and its three dimensions and by frequencies of the dimensions of the General Health Questionnaire (GHQ-28) of the total sample. As well as the values of Cronbach’s Alpha for each of the variables studied.

**Table 2:** Descriptive statistics of self-perceived stress, prenatal distress and general health.

	Questionnaire		*n* = 297	min	max	*M*	ST	*α*
PSS		Total	N/A	4	44	22.01	7.39	0.83
PDQ		Total	N/A	0	44	18.46	8.36	0.80
		PN	N/A	0	23	10.36	4.35	0.63
		PI	N/A	0	12	5.01	3.05	0.76
		PE	N/A	0	12	3.09	2.84	0.69
GHQ-28	SS							0.64
		Positive	6 (2%)	N/A	N/A	N/A	N/A	
		Negative	291 (98%)	N/A	N/A	N/A	N/A	
	AI							0.89
		Positive	54 (18.2%)	N/A	N/A	N/A	N/A	
		Negative	243 (81.8%)	N/A	N/A	N/A	N/A	
	SD							0.72
		Positive	72 (24.2%)	N/A	N/A	N/A	N/A	
		Negative	225 (75.8%)	N/A	N/A	N/A	N/A	
	*D*							0.88
		Positive	3 (1%)	N/A	N/A	N/A	N/A	
		Negative	294 (99%)	N/A	N/A	N/A	N/A	

min, minimum; max, maximum; M, Mean; ST, Standard Deviation; α, Cronbach’s Alpha; PN, Preoccupations about birth and baby; PI, Concerns about weight/personal image; PE, preoccupation with emotions SS, Somatic symptoms; AI, Anxiety-Insomnia; SD, Social dysfunction; D, Severe Depression; N/A, not applicable.

The main differences in parity (multiparous and primiparous) during the first trimester of pregnancy are shown in Table 3. The results showed that primiparous women presented higher levels of concern about childbirth and the baby (10.93 ± 4.73) than multiparous (9.88 ± 3.96). On the other hand, women who have no previous children presented higher levels of concern about for childbirth and the baby (10.79 ± 4.42) than the rest of the women (9.40 ± 4.07).

**Table 3:** Descriptive statistics of prenatal concerns according to parity, abortions, cesarean, and childbirth.

Questionnaire				min	max	*M*	Me	IQR	SD	*R*	MR	*p*
PDQ	TOTAL	Parity	Multiparous	1	42	18.14	16	11	8.05	41	144.44	0.315
			Primiparous	0	44	18.85	18	10	8.73	44	154.48	
		Abortions	None	0	44	18.44	17	11	8.30	44	149.82	0.801
			One or more	1	42	18.51	16	11	8.55	41	147.06	
		Cesarean	None	0	44	18.16	17	10	8.36	44	145.83	0.117
			One or more	5	38	20.21	15.5	12	8.18	33	168.24	
		Childbirth	None	0	44	18.66	17	11	8.36	44	152.15	0.342
			One or more	3	42	18.03	16	12	8.38	39	141.88	
	PN	Parity	Multiparous	1	21	9.88	9	5	3.96	20	139.15	0.030^*^
			Primiparous	0	23	10.93	10	6	4.73	23	160.83	
		Abortions	None	0	23	10.44	10	5	4.43	23	150.92	0.551
			One or more	1	21	10.16	10	6	4.18	20	144.43	
		Cesarean	None	0	23	10.26	10	6	4.43	23	146.67	0.248
			One or more	3	21	10.98	11	5	3.83	18	163.15	
		Childbirth	None	0	23	10.79	10	5	4.42	23	157.72	0.008^*^
			One or more	1	20	9.40	9	6	4.07	19	129.25	
	PI	Parity	Multiparous	0	12	5.25	5	4	3.15	12	154.45	0.229
			Primiparous	0	12	4.73	4	5	2.91	12	142.46	
		Abortions	None	0	12	4.92	5	5	2.97	12	147.17	0.569
			One or more	0	12	5.23	4	12	3.25	12	153.35	
		Cesarean	None	0	12	4.84	4	5	3.02	12	144.33	0.020^*^
			One or more	1	12	6.05	6.5	5	3.08	11	177.33	
		Childbirth	None	0	12	4.77	4	4	2.98	12	143.04	0.07
			One or more	0	12	5.55	5	5	3.17	12	1.62.49	
	PE	Parity	Multiparous	0	12	3.01	2	4	2.81	12	146.72	0.612
			Primiparous	0	12	3.19	2	4	2.89	12	151.74	
		Abortions	None	0	12	3.08	2	4	2.80	12	149.31	0.924
			One or more	0	12	3.12	2	4	2.97	12	148.27	
		Cesarean	None	0	12	3.06	2	4	2.83	12	148.16	0.676
			One or more	0	10	3.29	3	4	2.98	10	154.08	
		Childbirth	None	0	12	3.10	2	4	2.82	12	149.33	0.92
			One or more	0	12	3.09	2	4	2.91	12	148.25	

min, minimum; max, maximum; M, Mean; Me, Median; SD, Standard Deviation; IQR, interquartile ranges; R, Range; MR, Mena range; PN, Preoccupations about birth and baby; PI, Concerns about weight/personal image; PE, preoccupation with emotions. Significant interactions (*p* < 0.05) according to the Mann–Whitney *U* test.

On the other hand, we can observe, also in Table 3, that for concerns about weight/personal image, women who had had a previous cesarean section scored higher on this variable (6.05 ± 3.08) than those who did not undergone this experience (4.84 ± 3.02).

Table 4 presents the distribution of GHQ-28 scores in the study population according to parity. Of the participating women, 6% presented somatic symptoms according to the GHQ-28 scale, and all of them belong to the multiparous group. Regarding the anxiety/insomnia variable, 18% of the women scored positively (Table 2), with a higher percentage in multiparous women than in primiparous women (24.7% vs. 11.1%), as shown in Table 4. For social dysfunction and depression variables, no statistically significant differences were found in the frequencies between multiparous and primiparous women, although the frequency was higher in the multiparous group.

**Table 4:** Prevalence of General Health Questionnaire.

Questionnaire/variable		Primiparous (*n* = 136)	Multiparous (*n* = 162)	*χ^2^*	*p*
GHQ-28	SS			5.103	0.024^*^
	Positive	0 (0%)	6 (3.7%)		
	Negative	135 (100%)	156 (96.3)		
	AI			10.152	0.001^**^
	Positive	14 (11.1%)	40 (24.7%)		
	Negative	121 (88.9%)	122 (75.3%)		
	SD			0.221	0.639
	Positive	31 (22.8%)	41 (25.3%)		
	Negative	121 (77.2%)	104 (74.7%)		
	*D*			2.526	0.112
	Positive	0 (0%)	3 (1.8%)		
	Negative	135 (100%)	159 (98.2%)		

SS. Somatic symptoms; AI, Anxiety-Insomnia; SD, Social dysfunction; D, Severe Depression, *χ*2, Chi2 Square test; ^*^p < 0.05, ^**^p < 0.01.

When the Chi-square test was used to check for statistical independence, there was statistically significant evidence of a relationship between the variables somatic symptoms of psychological origin and anxiety/insomnia and the parity variable, as shown in Table 4.

In the Spearman’s correlation analysis (Table 5), positive correlations were found among the study variables, with statistically significant values among many of the study variables (*p* < 0.05). Thus, health perception was worse (higher values of somatic symptoms, anxiety/insomnia, social dysfunction and depression) when general stress and prenatal stress scores (concerns about birth and baby, weight/personal image, and emotions) were higher, and vice versa, as shown in Table 5.

**Table 5:** Spearman inter-scale correlations between all variables included in the present study.

	SS	AI	SD	*D*	PSS	PDQ	PN	PI
AI	0.118^*^	1						
	0.041							
SD	0.30	0.161^**^	1					
	0.601	0.005						
D	0.225^**^	0.214^**^	0.21	1				
	<0.001	<0.001	0.713					
PSS	−0.060	0.018	0.057	0.052	1			
	0.306	0.756	0.327	0.375				
PDQ	0.175^**^	0.262^**^	0.059	0.126^*^	0.07	1		
	0.003	<0.001	0.307	0.030	0.907			
PN	0.175^**^	0.184^**^	0.030	0.119^*^	0.050	0.827^**^	1	
	0.003	0.001	0.602	0.041	0.392	<0.001		
PI	0.138^*^	0.185^**^	0.026	0.105	−0.040	0.763^**^	0.399^**^	1
	0.018	0.001	0.652	0.072	0.493	<0.001	<0.001	
PE	0.128^*^	0.315^**^	0.094	0.126^*^	0.024	0.773^**^	0.510^**^	0.474^**^
	0.027	<0.001	0.104	0.030	0.676	<0.001	<0.001	<0.001

^*^p < 0.05, ^**^p < 0.01; SS, Somatic symptoms of psychological origin; AI, Anxiety-Insomnia; SD, Social dysfunction; D, Depression; PN, Preoccupations about birth and baby; PI, Concerns about weight/personal image; PE, preoccupation with emotions.

The results of the simple logistic regression analysis for the risk estimate (OR) are presented in Table 6. For each point in the assessment of prenatal concerns, the possibility of suffering somatic symptoms of psychological origin increased by 1.17 times. Regarding the dimensions of the PDQ, we observed that for each additional point in the evaluation of concerns about the birth of the baby, concerns about weight and personal image, and concerns about emotions, the possibility of experiencing somatic symptoms of psychological origin increased by 1.29, 1.60, and 1.40 times, respectively. In terms of self-perceived stress, no statistical significance was observed in risk estimation. However, we observed that for each point in the assessment of prenatal concerns, the possibility of suffering from anxiety and insomnia increased. Finally, we observed that for each point in the assessment of prenatal concerns, concerns about weight and personal image, and concerns about emotions, the possibility of suffering from depression increased by 1.18, 1.62, and 1.44 times, respectively.

**Table 6:** Logistic regression analysis between general health and prenatal distress.

Somatic symptoms					
		*R* ^2^	O.R.	95% IC	*p*
PDQ		0.040	1.17	1.06–1.29	0.001^*^
	PN	0.026	1.29	1.08–1.55	0.006^*^
	PI	0.036	1.60	1.16–2.19	0.004^*^
	PE	0.021	1.40	1.08–1.74	0.010^*^
PSS		0.002	0.95	0.85–1.07	0.400
*Anxiety-Insomnia*					
PDQ		0.071	1.09	1.05–1.13	<0.001^*^
	PN	0.027	1.10	1.03–1.18	0.004^*^
	PI	0.041	1.19	1.08–1.31	<0.001^*^
	PE	0.098	1.33	1.20–1.47	<0.001^*^
PSS		0.000	1.00	0.96–1.04	0.891
*Social dysfunction*					
PDQ		0.002	1.01	0.98–1.05	0.413
	PN	0.001	1.02	0.56–1.08	0.617
	PI	0.000	1.02	0.93–1.12	0.713
	PE	0.005	1.06	0.97–1.16	0.213
PSS		0.002	1.01	0.98–1.05	0.446
*Severe Depression*					
PDQ		0.022	1.18	1.03–1.34	0.017^*^
	PN	0.012	1.27	0.99–1.64	0.06
	PI	0.020	1.62	1.03–2.54	0.035^*^
	PE	0.015	1.44	1.03–2.02	0.035^*^
PSS		0.001	1.06	0.89–1.27	0.502

*R*^2^, Cox and Snell’s *R*^2^; PN, Preoccupations about birth and baby; PI, Concerns about weight/personal image; PE, preoccupation with emotions.

## Discussion

The present study focused on describing self-perceived health status, general stress, and prenatal stress, and on analyzing relationships and associations with sociodemographic factors.

The participants in the study presented a mean prenatal stress score (PDQ) of 18.46. This result was slightly higher than those found in similar studies, such as Awad-Sirhan et al. which was 16.98 (19) and with the results of Romero-Gonzalez et al. which was 16.87 (14), although lower than that present in other similar study, which was 23.45 (22), these three studies, like the present study belong to COVID-19 phase research. No statistically significant difference was observed between the mean PDQ analyzed. Values in the previous abortion group. This contrasts with the results found in a pre-COVID study in which they observed a mean PDQ value of 18.03 in women who had suffered any abortion and 12.95 in those who had not suffered any abortion (34). The populations of the aforementioned studies, as well as ours, belong to healthy women, which may explain these results.

Regarding self-perceived stress (PSS), the participants presented a mean score of 22.01, a lower value than in similar studies. In the study by Garcia-Silva et al. (22), the mean PSS score was 25.60 and in the study by Kashanian et al. (35), it was 25.5. However, as described by Karatas Baran et al. (36), which was 21, all of them were performed during the COVID-19 period. No statistically significant differences were found in the mean global stress values measured using the PSS as a function of parity, abortions, cesarean sections or previous children. However, other studies have found that primiparous pregnant women experience more stress or that multiparous women experience higher levels of stress (19, 26). The women in the present study were healthy and pregnant in the first trimester of gestation, which justifies these low levels of stress.

The variable “Concerns about the birth and the baby” presents a high mean score in relation to the maximum possible score, a result that is consistent with that presented in the research of Taubman – Ben Ari et al. (37). In the case of this variable “Concerns about the birth and about the baby” according to the subgroups parity, abortions and childbirth, we observed differences in the score between primiparous and multiparous women and between women with and without children, being higher in the group of primiparous women and in those without previous children in correspondence with similar studies that support that having given birth previously provides a protective factor against prenatal concern (5, 19, 26). In the case of previous abortions, a clear difference is also observed between the mean score of women without previous abortions and those who have had two or more, being higher in the latter, this case is similar to the results found in the study of Haghparast et al. (34).

We found a relatively low prevalence of the variable “Anxiety-Insomnia” (18.2%), which was much lower than the dare obtained in a similar study in which the prevalence of insomnia was 27.9 and 33.3% for anxiety, this may be because their study is not exclusive to women in the first trimester of pregnancy, our participants are all in the first trimester of gestation with an average of 8.7 weeks of gestation compared to 26.6 weeks of gestation of the study of Palagini et al. (38). Regarding anxiety, we found in another study that a quarter of the pregnant women presented moderate or severe anxiety and stress; this study was carried out in the COVID period (39). Another research also presents high prevalence regarding insomnia, presenting that 33.2% of their population presented this symptom (40). Despite these differences in relation to the prevalence of insomnia with our data, both Palagine et al. and Sanchez et al. (38, 40), affirm the relationship between pregnancy stress and the presence of insomnia, results that are comparable to those of our study. On the other hand, the results show a higher prevalence of insomnia-anxiety (24.7%) in the multiparous group than the primiparous group (11.1%), this is similar to the results regarding anxiety from the research of Dencker et al. (41). The other variable that shows a significantly higher prevalence in the multiparous group is “Somatic symptomatology,” which despite having a low prevalence in the total sample (2%) all of them are multiparous women, although the present study does not analyze pre-pandemic data, different baseline studies point to increased values of mental symptoms in the pregnant population due to the situation caused by the pandemic (8, 42, 43).

We cannot forget that measures must be taken to address the mental health of pregnant women and to carry out programs to overcome and alleviate stress during pregnancy (39, 44).

## Limitations

The limitations of this study should be considered when interpreting data. First, we may have a selection bias because we did not do a probability sampling Furthermore, all the subjects we recruited were from a specific area of northwestern Spain, and came from a regional hospital with a population of healthy people. The findings cannot be extrapolated to the entire population, as the present study aims to study this specific population. Second, the study research was based solely on quantitative data collected through online methods by means of self-administered questionnaires, and certain information biases could be identified as the administration of the questionnaire could not be assured. Finally, the sample size and subgroups obtained should be mentioned as limitations.

## Conclusion

During the first trimester of gestation, the pregnant women in our study presented high scores of stresses on the different scales used. Primiparous, women with previous abortions and those with no previous children had higher levels of concern about the pregnancy and the baby than the rest. Women with previous cesarean sections also had higher scores on Concerns about weight/personal image than the rest of the women. Multiparous women had a higher prevalence of somatic symptoms and more anxiety and insomnia than primiparous women. There is also a clear relationship between prenatal worries, anxiety, insomnia and depression with stress.

Health education that focuses on the mental health of pregnant women would help reduce worries during pregnancy and improve the pregnant women perception of her health and thus her well-being.

## Data availability statement

The raw data supporting the conclusions of this article will be made available by the authors, without undue reservation.

## Ethics statement

The studies involving human participants were reviewed and approved by Ethics committee of University of León (approval number: ETICA-ULE-033-2021) and of the Clinical Research Ethics Committee of the León and Bierzo Health Areas (approval number: 21124). The patients/participants provided their written informed consent to participate in this study.

## Author contributions

RG-F and CL-P: conceptualization and methodology. RG-F: formal analysis, investigation, and writing-original draft preparation. RG-F, MM-F, CM-V, and EF-M: writing-review and editing. CL-P, PH-L, and EF-M: supervision. All authors contributed to the article and approved the submitted version.

## Conflict of interest

The authors declare that the research was conducted in the absence of any commercial or financial relationships that could be construed as a potential conflict of interest.

## Publisher’s note

All claims expressed in this article are solely those of the authors and do not necessarily represent those of their affiliated organizations, or those of the publisher, the editors and the reviewers. Any product that may be evaluated in this article, or claim that may be made by its manufacturer, is not guaranteed or endorsed by the publisher.
